# Converting non-Mesogenic to Mesogenic Stacking of Amino-*s*-Triazine-Based Dendrons with *p*-CN Phenyl Unit by Eliminating Peripheral Dipole

**DOI:** 10.3390/nano12020185

**Published:** 2022-01-06

**Authors:** Yao-Chih Lu, Yu-Tsz Hsu, Tsung-Yen Yang, I-Chun Liou, Sheng-Wei Wang, Po-Chia Huang, Jey-Jau Lee, Long-Li Lai, Hsiu-Fu Hsu

**Affiliations:** 1 Department of Applied Chemistry, National Chi Nan University, No. 1 University Rd., Puli, Nantou 545, Taiwan; s103324901@mail1.ncnu.edu.tw (Y.-C.L.); s107324508@mail1.ncnu.edu.tw (T.-Y.Y.); s97324528@ncnu.edu.tw (I.-C.L.); yutze0301@gmail.com (S.-W.W.); 2 Department of Chemistry, Tamkang University, No. 151, Yingzhuan Rd., New Taipei City 251, Taiwan; s108324516@mail1.ncnu.edu.tw; 3 National Synchrotron Radiation Research Center, No. 101, Hsin-Ann Rd., Hsinchu 300, Taiwan; huang.pc@nsrrc.org.tw (P.-C.H.); jjlee@nsrrc.org.tw (J.-J.L.)

**Keywords:** triazine, liquid crystal, dendron

## Abstract

Three new amino-*s*-triazine-based dendrons, **1a**, **1b**, and **1c**, containing an aryl-CN moiety in the dendritic skeleton were prepared in 72–81% yields (**1a**: R^1^ = − N(*n*-C_8_H_17_)_2_, R^2^ = *n*-OC_8_H_17_, **1b**: R^1^ = R^2^ = − N(*n*-C_8_H_17_)_2_, **1c**: R^1^ = − N(*n*-C_8_H_17_)_2_, R^2^ = − N(*n*-C_4_H_9_)_2_). Dendrons **1a** with N(*n*-C_8_H_17_)_2_ and *n*-OC_8_H_17_ peripheral substituents, surprisingly, did not show any mesogenic phase during the thermal process. However, non-mesogenic **1a** can be converted to mesogenic **1b** or **1c** by eliminating the peripheral dipole arising from the alkoxy substituent; dendron **1b** only comprising the same N(*n*-C_8_H_17_)_2_ peripheral groups showed a ~25 °C mesogenic range on heating and ~108 °C mesogenic range on cooling. In contrast, dendron **1c** possessing different N(*n*-C_m_H_2m+1_)_2_ (m = 8 versus m = 4) peripheral units, having similar stacking as **1b**, exhibited a columnar phase on thermal treatment, but its mesogenic range (~9 and ~66 °C on heating and cooling, respectively) was much narrower than that of **1b,** attributed to **1c**’s less flexible alkyl chains in the peripheral part of dendron. Dendron **1a** with the alkoxy substituent in the peripheral skeleton, creating additional dipole correspondingly, thus, leads to the dendritic molecules having a non-mesogenic stacking. Without the peripheral dipole for intermolecular side-by-side interaction, dendrons **1b** and **1c** exhibit a columnar phase on thermal treatment because of the vibration from the peripheral alkyl chain.

## 1. Introduction

Dendrimers, probably generated from the combination of peripheral branches and central core, have three-dimensional spatial arrangements and are interesting; their applications in various research areas have been extensively studied [1,2,3,4,5,6,7,8,9]. Dendrons, containing part of the branched dendritic skeleton, are analogous to dendrimers in peripheral structure; they also have the characteristic of monodispersity and possess peripheral functional groups for specific purposes as dendrimers. Particularly, multiple functionalities can also be installed onto the periphery of dendrons for exhibiting versatile properties [10,11,12,13,14,15,16,17]. Surprisingly, though dendrimers as mesogens in liquid crystals have been extensively investigated because of their uniform packing and non-grained boundaries in the LC states [18,19,20,21,22,23,24,25,26,27,28,29,30], dendrons in the related studies are rather limited [31,32,33,34,35]. Possibly, dendrimers are rather symmetrical in chemical structures, which is advantageous for the easy formation of columnar phases [36,37,38,39,40,41,42,43,44,45,46,47,48,49]. The symmetrical structural feature favoring mesophase formation of dendrimers was adopted for several dendrons which self-assembled into dendrimers through H-bond interaction [50,51,52,53,54,55,56].

**Scheme 1:**
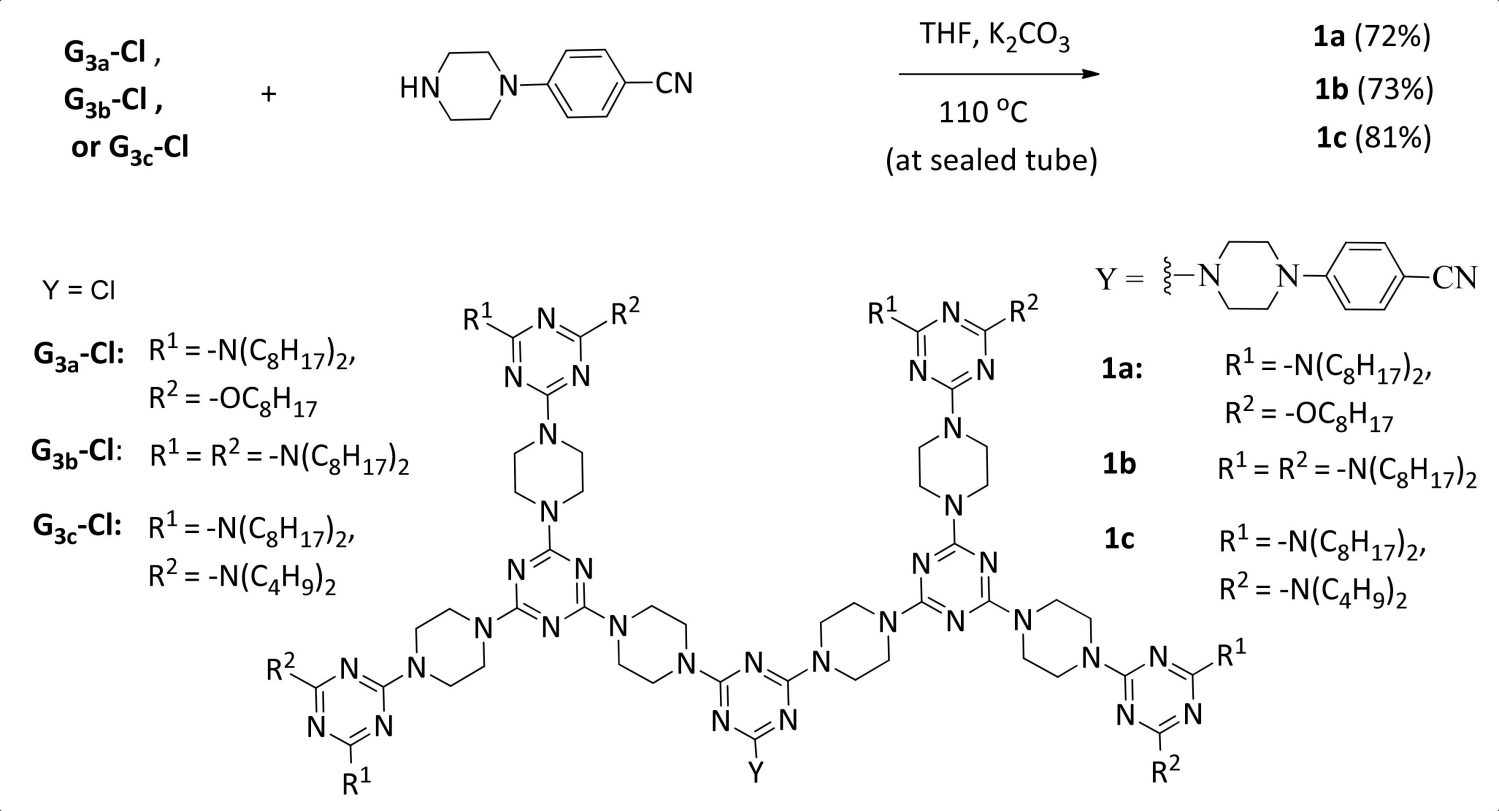
Preparation of dendrons **1a**–**c**.

We previously demonstrated that the addition of phenyl-CN lateral units in the central linker of dimeric G-3 dendrimers not only effectively broadened the range of columnar phase but also lowered the temperature of solidification on cooling [48], which can be important on the point of view of further practical applications. Discotic columnar (DC) liquid crystals possess good self-assembling ability in a long-range domain; DC materials with broad mesogenic range should be useful as solvating candidates in opto-electronic devices [23,54,57,58,59]. However, preparation of dimeric dendrimers with some particular functionality in the central linker may not be easy because of the steric effect from both dendronic halves. As we previously reported [48], the yields of the synthesis of dimeric dendrimers consisting of coupling two identical G-3 chloro-dendrons by means of *N*,*N*’-dibenzylpropane-1,3-diamine based central linker are only about 35%. Therefore, in order to simplify the synthesis and improve the yields of DC liquid crystals, we turn our attention to dendron-precursors, by introducing a strong polar CN functionality to the dendritic skeleton and thus hoping to lead the dendrons to exhibit a broader mesogenic range on thermal treatment. Previously, we also demonstrated that breaking the symmetry of the amino-*s*-triazine-based dendrimers by linking different peripheral groups in the same molecule successfully converted non-mesogenic to mesogenic dendrimers [44]. Based on the combined strategies, i.e., incorporation of a CN polarity and symmetry-breaking, we efficiently prepared dendrons **1a**–**1c** in 72–81% yields from the reaction of G-3 chloro-dendrons with 4-cyanophenylpiperazine (Scheme 1). Particularly, dendron **1b** showed a ~108 °C mesogenic range on cooling, which is better than the mesogenic ranges of most of the previously reported amino-*s*-triazine-based dendrimers on thermal treatment [43,44,45,46,47,48,49] and should possess potential application in the related opto-electronic devices. If so, compared with dendrimers, their dendron-precursors probably possess lower molecular weights and may be easier in preparation because significant steric effects generally arise from the both dendronic halves in preparing dendrimers. Based on the lower cost in chemicals and the better efficiency in preparation, it is worthwhile to prepare and study dendrons in their solid stackings. We herein wish to report the results.

## 2. Experimental Section

### 2.1. General Methods

Chemicals were used after purchase without further purification. Elemental analyses were performed using an Elementar Unicube analyzer (Elementar, Langenselbold, Germany). NMR spectra were recorded on Bruker AM 300 instrument (Bruker, Billerica, MA, USA) operating at 300 and 75 MHz for ^1^H and ^13^C nuclei, respectively. All chemical shifts (δ values) are given in parts per million (ppm); all homocoupling patterns (n, J, H, *H* values) are given in Hertz. Chemical shifts were measured against TMS as reference signal. The MALDI-TOF mass spectra were obtained from a Voyager-DE PRO (Applied Biosystems) mass spectrometer (Bruker, Billerica, MA, USA). The mesogenic textures and phase transitions were obtained from polarizing optical microscopy and differential scanning calorimetry (Perkin-Elmer DSC Diamond; Perkin Elmer, Waltham, MA, USA). The XRD studies were performed at the temperature of mesogenic ranges. Synchrotron power X-ray diffraction (XRD) measurements were completed at the National Synchrotron Radiation Research Center (NSRRC), Hsinchu, Taiwan, with the X-ray wavelength of 1.334431 Å. The powder samples were added into a capillary tube, and the temperature controller is programmable by a PC with a PID feedback system.

### 2.2. Sample Characterization by POM

4-{4-{4,6-Bis{4-{4,6-bis{4-{[4-di(n-octyl)amino,6-(n-octyloxy)]-s-triazin-2-yl}-piperazin-1-yl}-s-triazin-2-yl}-piperazin-1-yl}-s-triazin-2-yl}-piperazin-1-yl}-benzonitrile **1a** was heated to 210 °C to let **1a** become isotropic, and then cooled to room temperature at the rate of 20 °C/min in the first thermal cycle. Compound **1a** was further treated at the rate of 5 °C/min on heating, and then 1 °C/min on cooling in the second cycle. 4-{4-{4,6-Bis{4-{4,6-bis{4-{[4-di(n-octyl)amino,6-di(n-octyl)]-s-triazin-2-yl}-piperazin-1-yl}-s-triazin-2-yl}-pip-erazin-1-yl}-s-triazin-2-yl}-piperazin-1-yl}-benzonitrile **1b** and 4-{4-{4,6-Bis{4-{4,6-bis{4-{[4-di(n-octyl)amino,6-di(n-butyl)]-s-triazin-2-yl}-piperazin-1-yl}-s- triazin-2-yl}-piperazin-1-yl}-s-triazin-2-yl}-piperazin-1-yl}-benzonitrile **1c** were treated similarly.

### 2.3. Sample Characterization by DSC

**1a** was heated to 210 °C, and then cooled to room temperature at the rate of 10 °C/min in the first cycle. In the second thermal process, **1a** was then heated and cooled at the rate of 5 °C/min for recording the transition temperature and corresponding enthalpies. **1b** and **1c** were accordingly treated.

### 2.4. Prepartion of Chloro-Dendrons G_3a_-Cl and G_3c_-Cl

**G_1c_-Cl:** Cyanuric chloride (3.68 g, 20.0 mmol) was added to dry DCM (100 mL) at 5 °C, and dioctylamine (4.83 g, 20.0 mmol) in dry DCM (10.0 mL) was then added. The reaction mixture was stirred for 30 min. After the reaction was confirmed to be complete by silica-TLC, KOH (3.37 g, 60.0 mmol) in water (50 mL) was added. After stirring, the organic solvent was separated, dried over MgSO_4_, and then followed by the addition of dibutylamine (2.58 g, 20.0 mmol) in dry DCM (10.0 mL). After 30 min, trimethylamine (6.07 g, 60.0 mmol) was added, and the reaction mixture was stirred at room temperature for 10 h. After the reaction was confirmed to be complete by silica-TLC, KOH (3.37 g, 60.0 mmol) in water (50 mL) was added. After stirring, the organic solvent was separated, dried over MgSO_4_, and then concentrated under reduced pressure to dryness to give a colorless liquid, which was purified by chromatography (15 × 2.1 cm of silica; eluates DCM/hexane (1/4 *v*/*v*)) to yield **G_1c_-Cl** as a colorless liquid in 83% yield (7.97 g) with respect to Cyanuric chloride. ^1^H NMR (300 MHz, CDCl_3_, 25 °C): δ_H_ 0.89 (t, *J* = 5.4 Hz, 6H, 2 × CH_3_), 0.93 (t, *J* = 5.4 Hz, 6H, 2 × CH_3_), 1.15 (s, br. 24H, 12 × CH_2_), 1.55 (s, br. 8H, 4 × CH_2_), 3.39–3.51 (m, 8H, 4 × CH_2_) ppm. ^13^C NMR (75 MHz, CDCl_3_, 25 °C): δ_C_ 20.2, 20.5, 22.8, 27.0, 27.4, 27.8, 28.2, 29.4, 29.5, 29.7, 29.9, 30.4, 32.0, 46.9, 47.2, 47.3, 47.6, 164.6, 169.1 ppm. MS calculated for C_27_H_53_N_5_Cl [M + H]^+^: 483.20; found 483.28. HRMS: calculated for C_27_H_53_N_5_^35^Cl [M + H]^+^: 482.3989; found 482.4003.

**G_1c_-N~NH: G_1c_-Cl** (15.53 g, 32.2 mmol) in THF (10mL) was added to piperazine (8.32 g, 96.6 mmol) in THF (100 mL), and the resulting solution was stirred at 40 °C for 30 min. Triethylamine (9.78 g, 96.6 mmol) was added, and the solution was stirred for another 15 h. After checking the completion of the reaction by silica-TLC, KOH (5.42 g, 96.6 mmol) in water (50 mL) was added. After stirring the mixture, the organic solvent was separated, dried over MgSO_4_, and then concentrated under reduced pressure to dryness to give a colorless liquid, which was purified by chromatography (10 × 2.1 cm of silica; eluates DCM/THF (4/1 *v*/*v*)) to yield **G_1c_-N~NH** as a colorless liquid in 80% yield (13.69 g) with respect to **G_1c_-Cl**. ^1^H NMR (300 MHz, CDCl_3_, 25 °C): δ_H_ 0.89 (t, *J* = 5.4 Hz, 6H, 2 × CH_3_), 0.93 (t, *J* = 5.4 Hz, 6H, 2 × CH_3_), 1.29 (s, br. 24H, 12 × CH_2_), 1.55–1.61 (m, 8H, 4 × CH_2_), 1.84 (s, 1H, NH), 2.87 (t, *J* = 5.1 Hz, 4H, 2 × CH_2_), 3.42–3.49 (m, 8H, 4 × CH_2_), 3.72–3.78 (m, 4H, 2 × CH_2_) ppm. ^13^C NMR (75 MHz, CDCl_3_, 25 °C): δ_C_ 14.2, 14.3, 20.5, 22.8, 25.8, 27.4, 28.3, 29.5, 29.7, 30.5, 32.0, 44.6, 46.2, 46.8, 47.1, 68.1, 125.7, 165.3, 165,5 ppm. MS calculated for C_31_H_62_N_7_ [M + H]^+^: 532.88; found 532.39. HRMS: calculated for C_31_H_62_N_7_ [M + H]^+^: 532.5067; found 532.5087.

**G_2c_-Cl: G_1c_-N~NH** (10.5 g, 19.8 mmol) in dry DCM (10.0 mL) was added to cyanuric chloride (1.82 g, 9.88 mmol) in dry DCM (50 mL) at 5 °C, and the reaction mixture was then stirred for 30 min. Triethylamine (6.07 g, 60.0 mmol) was added, and the reaction mixture was stirred at room temperature for 18 h. After the reaction was confirmed to be complete by silica-TLC, KOH (3.37 g, 60.0 mmol) in water (50 mL) was added. After stirring, the organic solvent was separated, dried over MgSO_4_, and then concentrated under reduced pressure to dryness to give a colorless residue, which was purified by chromatography (15 × 2.1 cm of silica; eluates DCM/hexane (1/2 *v*/*v*)) to yield **G_2c_-Cl** as a colorless solid in 62% yield (7.25 g) with respect to **G_1c_-N~NH**. ^1^H NMR (300 MHz, CDCl_3_, 25 °C): δ_H_ 0.85–0.94 (m, 24H, 8 × CH_3_), 1.36 (s, br. 48H, 24 × CH_2_), 1.53–1.59 (m, 16H, 8 × CH_2_), 3.42–3.48 (m,16H, 8 × CH_2_), 3.81 (s, br. 16H, 8 × CH_2_) ppm. ^13^C NMR (75 MHz, CDCl_3_, 25 °C): δ_C_ 14.2, 14.3, 20.5, 22.8, 27.4, 28.4, 29.5, 29.7, 30.5, 31.7, 32.0, 42.9, 43.2, 43.6, 46.8, 46.8, 47.2, 47.4, 164.7, 165.2, 165.2, 165.5, 169.8 ppm. MS calculated for C_65_H_120_N_17_Cl [M + H]^+^: 1176.22; found 1175.83. HRMS: calculated for C_65_H_121_N_17_^35^Cl [M + H]^+^: 1174.9697; found 1174.9697.

**G_2c_-N~NH** (6.63 g, 88%), with respect to **G_2c_-Cl**, was obtained from the reaction of **G_2c_-Cl** (7.25 g, 6.16 mmol) with piperazine (1.59 g, 18.48 mmol) in a similar manner to prepare **G_1c_-N~NH**. ^1^H NMR (300 MHz, CDCl_3_, 25 °C): δ_H_ 0.85–0.95 (m, 24H, 8 × CH_3_), 1.25 (s, 48H, 24 × CH_2_), 1.56 (s, 16H, 8 × CH_2_), 1.99 (s, 1H, NH), 2.89 (s, br. 4H, 2 × CH_2_), 3.45 (s, br. 16H, 8 × CH_2_), 3.78 (s, 20H, 10 × CH_2_) ppm. ^13^C NMR (75 MHz, CDCl_3_, 25 °C): δ_C_ 14.2, 14.3, 20.5, 22.8, 25.7, 27.4, 28.4, 29.5, 29.6, 30.5, 31.7, 32.0, 43.3, 44.5, 46.2, 47.1, 68.1, 165.3, 165.6, 165.6 ppm. MS calculated for C_69_H_129_N_19_ [M]^+^: 1224.89; found 1224.37. Elemental analysis calculated for C_69_H_129_N_19_: C 67.66; H 10.62; N 21.73; found C 67.99 H 10.68; N 21.60.

**G_3c_-Cl** (0.78 g, 63%), with respect to **G_2c_-N~NH**, was obtained from the reaction of **G_2c_-N~NH** (1.19 g, 0.97 mmol) with cyanuric chloride (0.09 g, 0.48 mmol) in a similar manner to prepare **G_2c_-Cl**. ^1^H NMR (300 MHz, CDCl_3_, 25 °C): δ_H_ 0.86–0.96 (m, 48H, 16 × CH_3_), 1.28 (s, br. 96H, 48 × CH_2_), 1.57 (s, br. 32H, 16 × CH_2_), 3.46 (s, br. 32H, 16 × CH_2_), 3.79+3.83 (2s, 48H, 24 × CH_2_) ppm. ^13^C NMR (75 MHz, CDCl_3_, 25 °C): δ_C_ 14.2, 20.5, 22.8, 27.3, 28.3, 29.5, 29.7, 30.5, 32.0, 43.2, 43.3, 43.6, 47.0, 47.3, 164.7, 165.3, 165.5 ppm. MS calculated for C_141_H_256_N_41_Cl [M]^+^: 2561.27; found 2561.47. Elemental analysis calculated for C_141_H_256_N_41_Cl: C 66.12; H 10.17; N 22.42; found C 66.38 H 10.15; N 22.21.

**G_3a_-Cl** (2.13 g, 76%), with respect to **G_2a_-N~NH**, was obtained from the reaction of **G_2c_-N~NH** (2.50 g, 2.0 mmol) with cyanuric chloride (0.18 g, 1.0 mmol) in a similar manner to prepare **G_2c_-Cl**. ^1^H NMR (300 MHz, CDCl_3_, 25 °C): δ_H_ 0.89 (s. br., 36H, 12 × CH_3_), 1.29 (s, br. 120H, 60 × CH_2_), 1.60 (s, br. 16H, 8 × CH_2_), 1.72–1.81 (m, 8H, 4 × CH_2_), 3.46 (s, br. 16H, 8 × CH_2_), 3.82 (s, 48H, 24 × CH_2_), 4.28 (t, *J* = 6.9 Hz, 8H, 4 × CH_2_) ppm. ^13^C NMR (75 MHz, CDCl_3_, 25 °C): δ_C_ 14.1, 22.6, 26.0, 27.1, 27.7, 28.1 29.0, 29.2, 29.3, 29.4, 31.8, 43.1, 47.1, 66.6, 165.4, 165.9, 166.2, 177.7 ppm. MS calculated for C_141_H_251_N_37_O_4_Cl [M − H]^+^: 2564.21; found 2564.00. Elemental analysis calculated for C_141_H_252_ N_37_O_4_Cl: C 66.02; H 9.90; N 20.20; found C 66.22 H 10.02; N 20.26.

### 2.5. The Typical Procedure for Preparing Dendrons **1a**–**1c**

To a THF (25 mL) solution containing dendron **G_3a_-Cl** (0.28 g, 0.10 mmol) and 4-piperazinobenzontrile (0.03 g, 0.15 mmol), K_2_CO_3_ (0.04 g, 0.30 mmol) was added, and the resulting suspension was then heated at 110 °C for 18 h in a sealed tube. After the reaction was confirmed to be complete by silica-TLC, the solvent was removed under reduced pressure to dryness, and then a water (50 mL) solution containing K_2_CO_3_ (0.14 g, 0.10 mmol) was added. The mixture was extracted with DCM (2 × 30 mL). The combined organic extracts were dried over MgSO_4_ and then concentrated under reduced pressure to dryness. The crude solid residue was recrystallized from a DCM/MeOH (20/1 *v*/*v*) mixture to give dendron **1a** as a colorless solid in 72% yield (0.23 g) with respect to G_2a_-Cl. ^1^H NMR (300 MHz, CDCl_3_, 25 °C): δ_H_ 0.88 (s br., 36H, 12×CH_3_), 1.29 (s br., 120H, 60 × CH_2_), 1.61 (s br., 16H, 8 × CH_2_), 1.71–1.75 (m, 8H, 4 × CH_2_), 3.37 (s, 4H, 2 × CH_2_), 3.49 (s, 16H, 8 × CH_2_), 3.82 (s, 48H, 24 × CH_2_), 3.94 (s, 4H, 2 × CH_2_), 4.27 (t, *J* = 4.8 Hz, 8H, 4 × CH_2_), 6.89 (d, *J* = 8.4 Hz, 2H, 2 × CH), 7.51 (d, *J* = 8.4 Hz, 2H, 2 × CH) ppm. ^13^C NMR (75 MHz, CDCl_3_, 25 °C): δ_C_ 14.3, 22.9, 26.3, 27.3, 27.9, 28.3, 29.2, 29.5, 29.6, 32.0, 43.4, 47.3, 66.8, 100.7, 114.5, 120.2, 133.8, 153.6, 165.6, 166.1, 166.4, 170.9 ppm. MS calculated for C_152_H_264_N_40_O_4_ [M]^+^: 2715.9; found 2716.0. Elemental analysis calculated for C_152_H_264_N_40_O_4_: C 67.22; H 9.80; N 20.63; found C 67.58 H 9.59; N 20.41.

Dendrons **1b** and **1c** were prepared accordingly from reactions of 4-piperazinobenzontrile (0.03g, 0.15 mmol) with **G_3b_-Cl** (0.30 g, 0.1 mmol) and **G_3c_-Cl** (0.27 g, 0.1 mmol), respectively.

Dendron **1b**: A colorless solid in 73% (0.20 g). ^1^H NMR (300 MHz, CDCl_3_, 25 °C): δ_H_ 0.88 (s br., 48H, 16 × CH_3_), 1.28 (s br., 160H, 80 × CH_2_), 1.58 (s br., 32H, 16 × CH_2_), 3.37 (s, 4H, 2 × CH_2_), 3.45 (s, 32H, 16 × CH_2_), 3.82 (s, 48H, 24 × CH_2_), 3.93 (s, 4H, 2 × CH_2_), 6.88 (d, *J* = 8.7 Hz, 2H, 2 × Ar-H), 7.52 (d, *J* = 8.7 Hz, 2H, 2 × Ar-H) ppm. ^13^C NMR (75 MHz, CDCl_3_, 25 °C): δ_C_ 14.4, 22.9, 27.4, 27.6, 28.4, 28.5, 29.5, 29.7, 29.9, 32.1, 43.4, 47.1, 47.4, 100.7, 114.5, 120.2, 133.8, 153.6, 165.3, 165.6 ppm. MS calculated for C_183_H_332_N_44_ [M]^+^: 3160.9; found 3160.8. Elemental analysis calculated for C_183_H_332_N_44_: C 69.80; H 10.60; N 19.57; found C 70.08; H 10.68; N 19.24.

Dendron **1c**: A colorless solid in 81% (0.23 g). ^1^H NMR (300 MHz, CDCl_3_, 25 °C): δ_H_ 0.86–0.96 (m, 48H, 16 × CH_3_), 1.27 (s br.,96H, 48 × CH_2_), 1.56 (s br., 32H, 16 × CH_2_), 3.37 (s br., 4H, 2 × CH_2_), 3.46 (s br., 32H, 16 × CH_2_), 3.80 + 3.83 (2s, 48H, 24 × CH_2_), 3.94 (s br., 4H, 2 × CH_2_), 6.88 (d, *J* = 8.4 Hz, 2H, 2 × Ar-H), 7.52 (d, *J* = 8.4 Hz, 2H, 2 × Ar-H). ^13^C NMR (75 MHz, CDCl_3_, 25 °C): δ_C_ 14.3, 20.6, 22.9, 27.4, 27.5, 28.4, 28.5, 29.6, 29.7, 29.8, 30.6, 32.1, 43.4, 46.8, 47.1, 47.4, 100.7, 114.5, 120.2, 133.8, 153.6, 165.3, 165.6 ppm. MS calculated for C_152_H_268_N_44_ [M]^+^: 2712.1; found 2712.8. Elemental analysis calculated for C_152_H_268_N_44_: C 67.32; H 9.96; N 22.72; found C 67.09 H 9.89; N 22.47. 

## 3. Results and Discussion

**Scheme 2:**
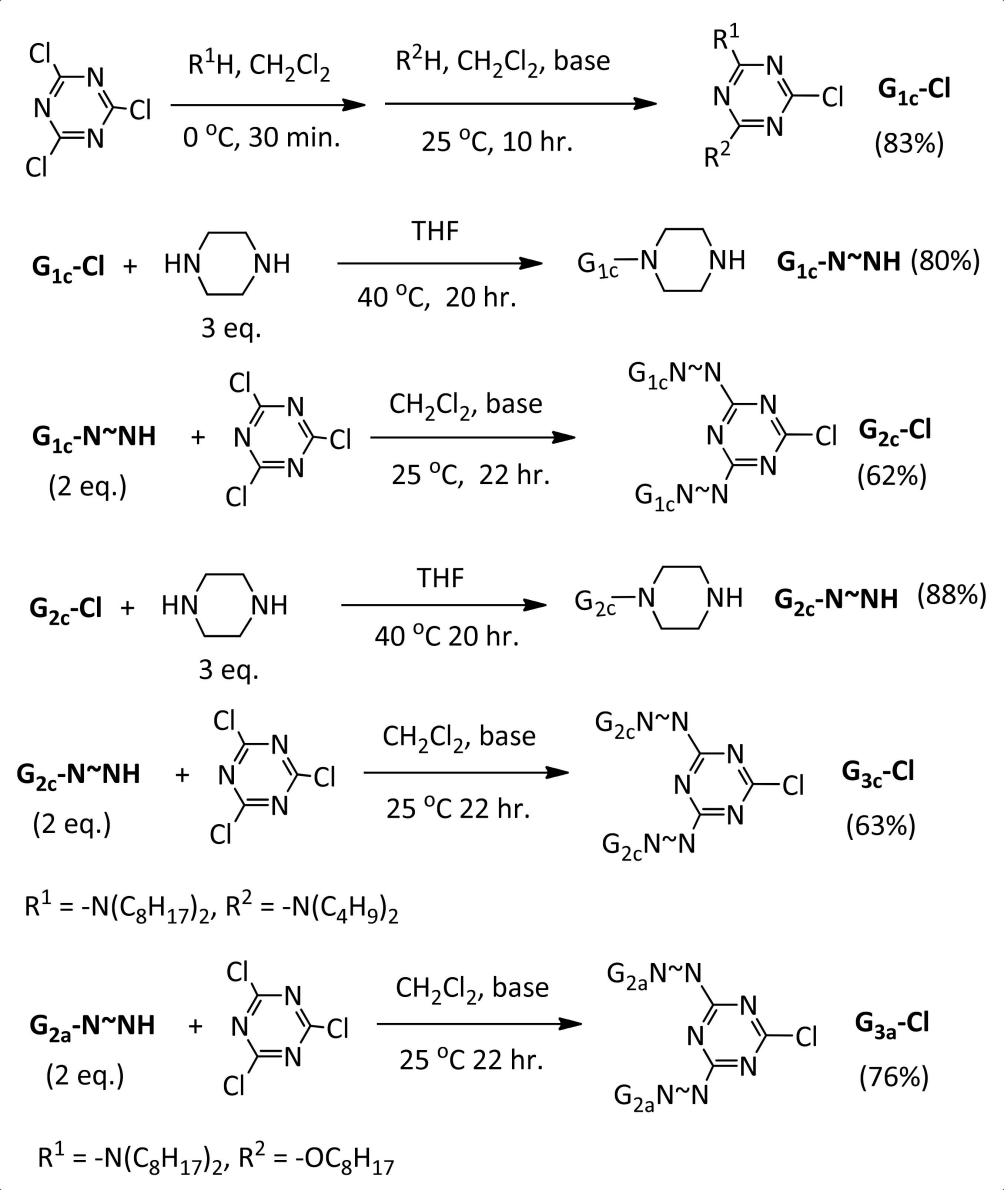
Preparation of compounds **G_3a_-Cl** and **G_3c_-Cl**.

We previously reported [43,44,45,46,47,48] the synthesis of G-2 piperazine-dendrons **G_2a_-N~NH** and **G_2b_-N~NH**, and G-3 chloro-dendrons **G_3b_-Cl**. **G_2c_-N~NH** was thus prepared in a similar manner, and, subsequently, reaction of **G_2a_-N~NH** and **G_2c_-N~NH** with cyanuric chloride produced **G_3a_-Cl** and **G_3c_-Cl**, respectively (Scheme 2). Reaction of **G_3a_-Cl**, **G_3b_-Cl**, and **G_3c_-Cl** with 4-piperazinobenzontrile produced dendrons **1a** (~72%), **1b** (~73%), and **1c** (~81%), respectively (Scheme 1). Dendrons **1a**,**b**,**c** were fully confirmed by ^1^H and ^13^C NMR spectroscopy, elemental analysis, and MALDI-TOF mass spectrometry.

**Figure 1:**
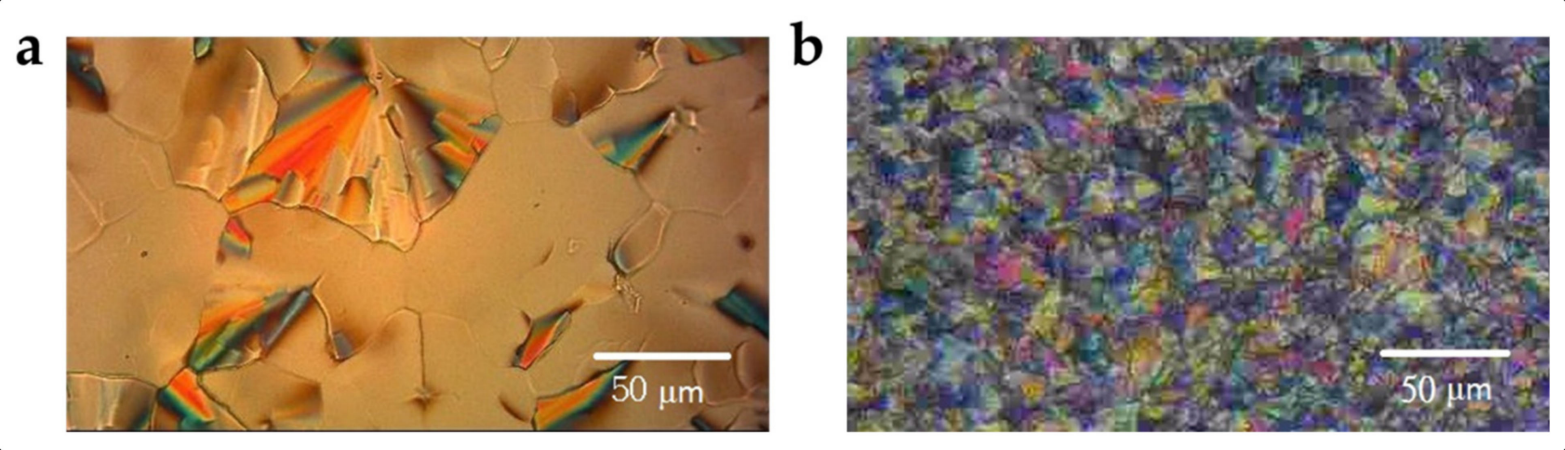
The POM textures of dendrons **1b** (**a**) and **1c** (**b**), which were taken at 180 °C and 165 °C, respectively, in the second cycle at the cooling rate of 1 °C/min.

**Scheme 3:**
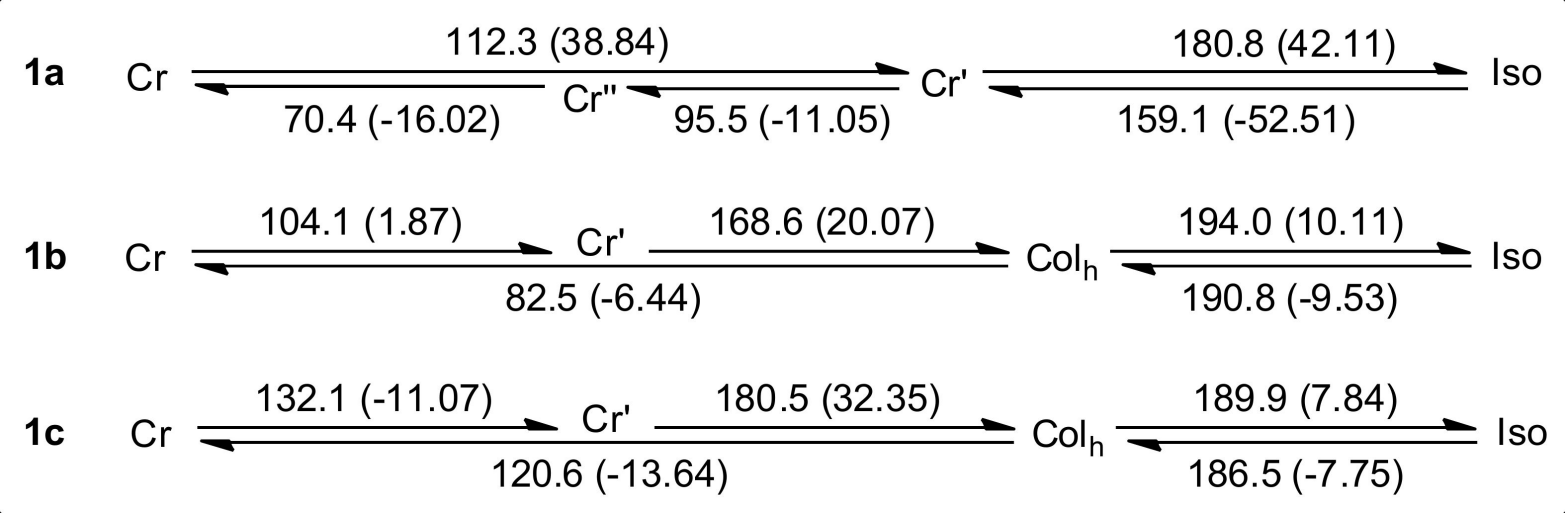
The enthalpies (kJ/mol) of dendrons **1a**–**1c** at various phase transition temperatures. Cr, Cr’, and Cr’’ all represent the crystalline state but in different packings. Col_h_ and Iso denote the hexagonal columnar and isotropic phases, respectively.

Dendron **1a** did not exhibit any mesogenic phase either on heating or on cooling. Dendrions **1b** and **1c** were observed to show a mosaic texture under polarizing optical microscopy (POM) on thermal treatment (Figure 1). The thermal behaviors of dendrons **1a**–**1c** were also examined by differential scanning calorimetry (DSC) (Figures S1–S3) and are summarized in Scheme 3. Surprisingly, dendron **1a** directly solidified at ~159 °C from the isotropic state without showing mesogenic phase on cooling, which was confirmed by the studies of POM (Figure S4) and seemed to differ from our previous observation that dendrimers with various peripheral functionalities exhibited better mesogenic ranges than those with identical peripheral functionalities [44]; this will be discussed in detail later. Dendron **1b** showed a hexagonal columnar mesophase between ~191 °C and ~83 °C on cooling. Dendron **1c** with different peripheral functionalities was, thus, further prepared for comparison; **1c** exhibited a hexagonal columnar mesophase between ~187 °C and ~121 °C on cooling. The Iso-to-Col_h_ transition temperature of **1b** and **1c** seems not to differ much from each other, but the mesogenic ranges for **1b** (~108 °C) is very different from that of **1c** (~66 °C) on cooling because of supercooling of **1b**. However, the mesogenic ranges on heating for **1b** (between ~169 and ~194 °C) and **1c** (between ~181 and ~190 °C) are not so much different.

**Figure 2:**
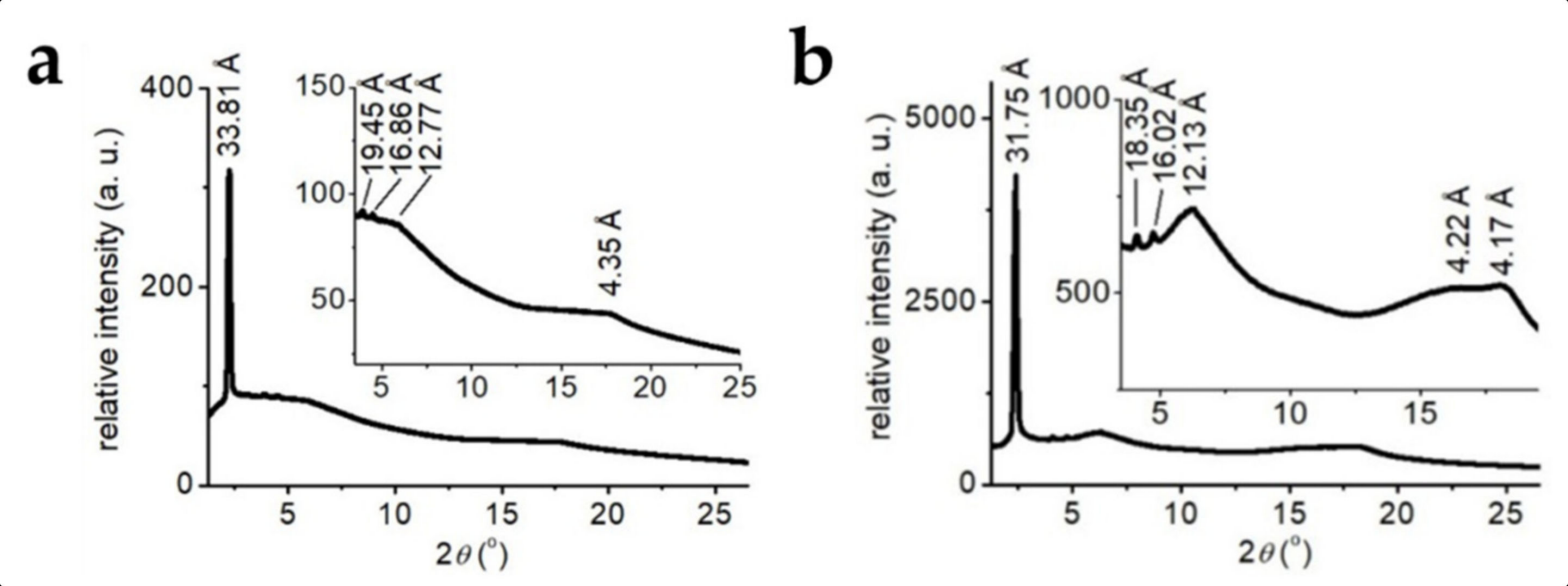
The reflections of powder-XRD of dendrons **1b** (**a**) and **1c** (**b**), which were recorded at 180 °C on heating and 165 °C on cooling, respectively.

The mesogenic phases of dendrons **1b** and **1c** were characterized to be hexagonal columnar by powder-XRD (Figure 2). A peak at 33.81 Å, which is sharp and appears in the small-angle region of powder XRD of **1b**, arose from the reflection of *d*10. The two weak signals at 19.45 and 16.86 Å resulted from the reflections of *d*11 (calculated 19.52 Å) and *d*20 (calculated 16.91 Å), respectively. The significantly larger linewidth of the broad signal at 12.77 Å than those of *d*10, *d*11, and *d*20 excludes the possibility of assigning the signal to be *d*21, though the d-spacing is close to the calculated *d*21 of 12.78 Å. The signal at 12.77 Å can be due to intracolumnar correlations based on its characteristic of the large line-width. The broad signal at 4.35 Å with the characteristic of a weak correlation was attributed to chain correlations. The XRD reflection pattern indicated that the calculated lattice constant *a* of dendron **1b** in the hexagonal columnar phase was 39.04 Å. The XRD reflection pattern of **1c** was similar to that of **1b**, indicating both dendrons have the same mesophases during the thermal process. The *d*10, *d*11, *d*20, and the lattice constant *a* of **1c** were 31.75, 18.35, 16.02, and 36.66 Å, respectively. For comparison, the calculated *d*11 and *d*20 were 18.33 and 15.88 Å, respectively. It is noteworthy that two broad wide-angle signals at 4.22 and 4.17 Å were observed for **1c**, showing the mixed stacking of alkyl halos from the weak correlations of two amino groups, i.e., N(*n*-C_8_H_17_)_2_ and N(*n*-C_4_H_9_)_2_, as shown in the literature [44].

**Figure 3:**
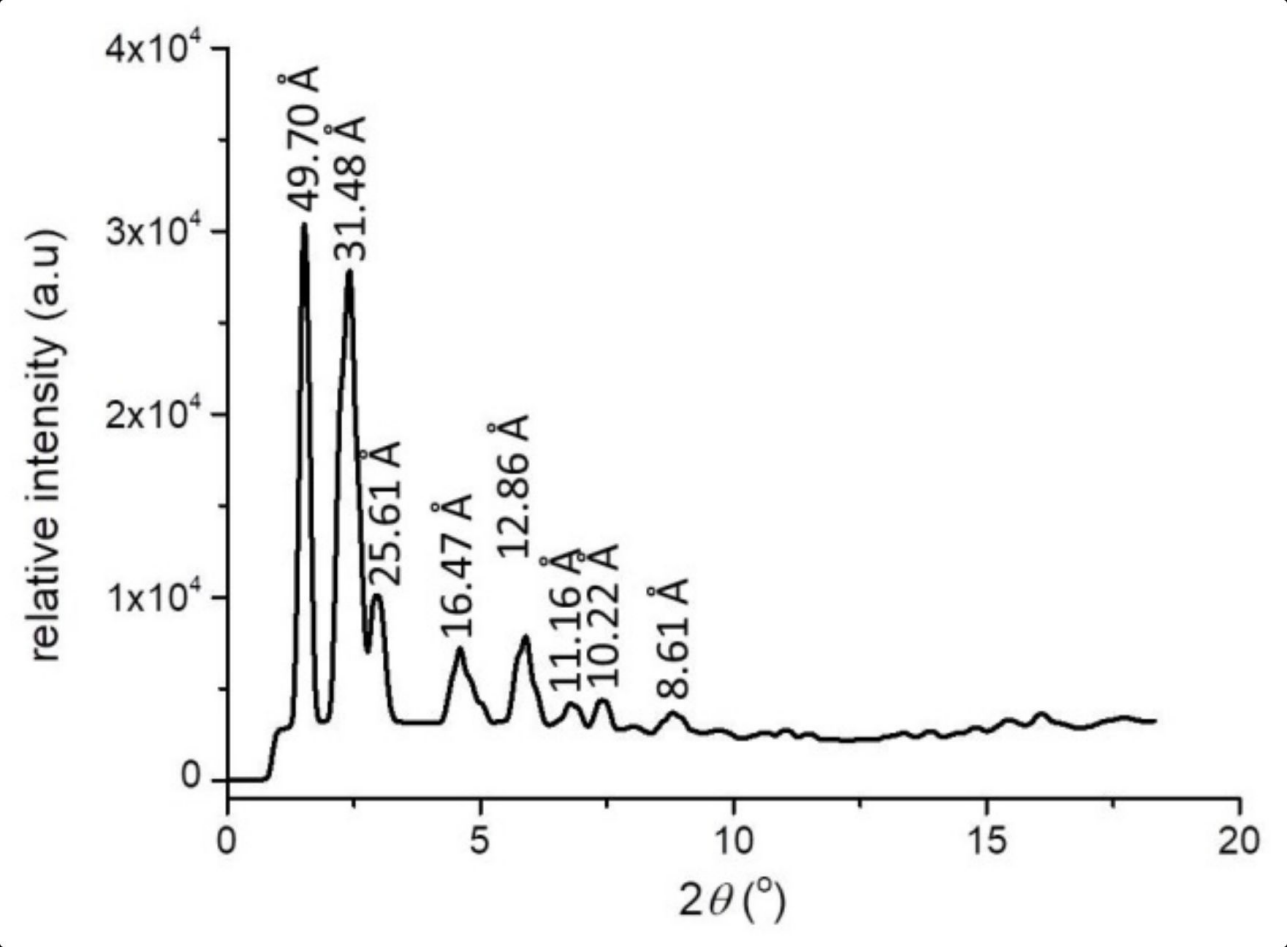
The powder-XRD of dendron **1a** at 145 °C on cooling.

Dendron **1a**, containing another constructing unit **IIC** with lower dipole moment in skeleton, was also studied by powder-XRD; based on the reflection pattern at 145 °C on cooling (Figure 3), it is reasonable to assume that dendron **1a** has a more highly ordered arrangement than **1b** and **1c** in the related temperature range, which is also supported by the DSC of **1a** (Figure S1).

Although, in a previous report [44], we noticed that linking different peripheral functionalities to the same dendritic skeleton can convert the non-mesogenic dendrimer to become mesogenic. This seems not to be applicable to the case of dendrons under current study. Indeed, dendron **1a** bearing N(*n*-C_8_H_17_)_2_ and *n*-OC_8_H_17_ peripheral substituent, did not exhibit any mesogenic behaviors during the thermal process. By contrast, having installed identical N(*n*-C_8_H_17_)_2_ (**1b**) or different N(*n*-C_8_H_17_)_2_ versus N(*n*-C_4_H_9_)_2_ (**1c**) substituents at the periphery, dendrons **1b** and **1c** were observed to be mesogenic on thermal treatment. To have a deeper understanding of such the observation, the conformations of dendrons **1a**–**c** were studied by CaChe using the MM2 model. The conformation of **1b** was obtained by combining the optimized dendron **G_3b_-Cl** [47] with 4-piperazinobenzonitrile and then optimized. The conformations of **1a** and **1c** were optimized in a similar manner.

**Figure 4:**
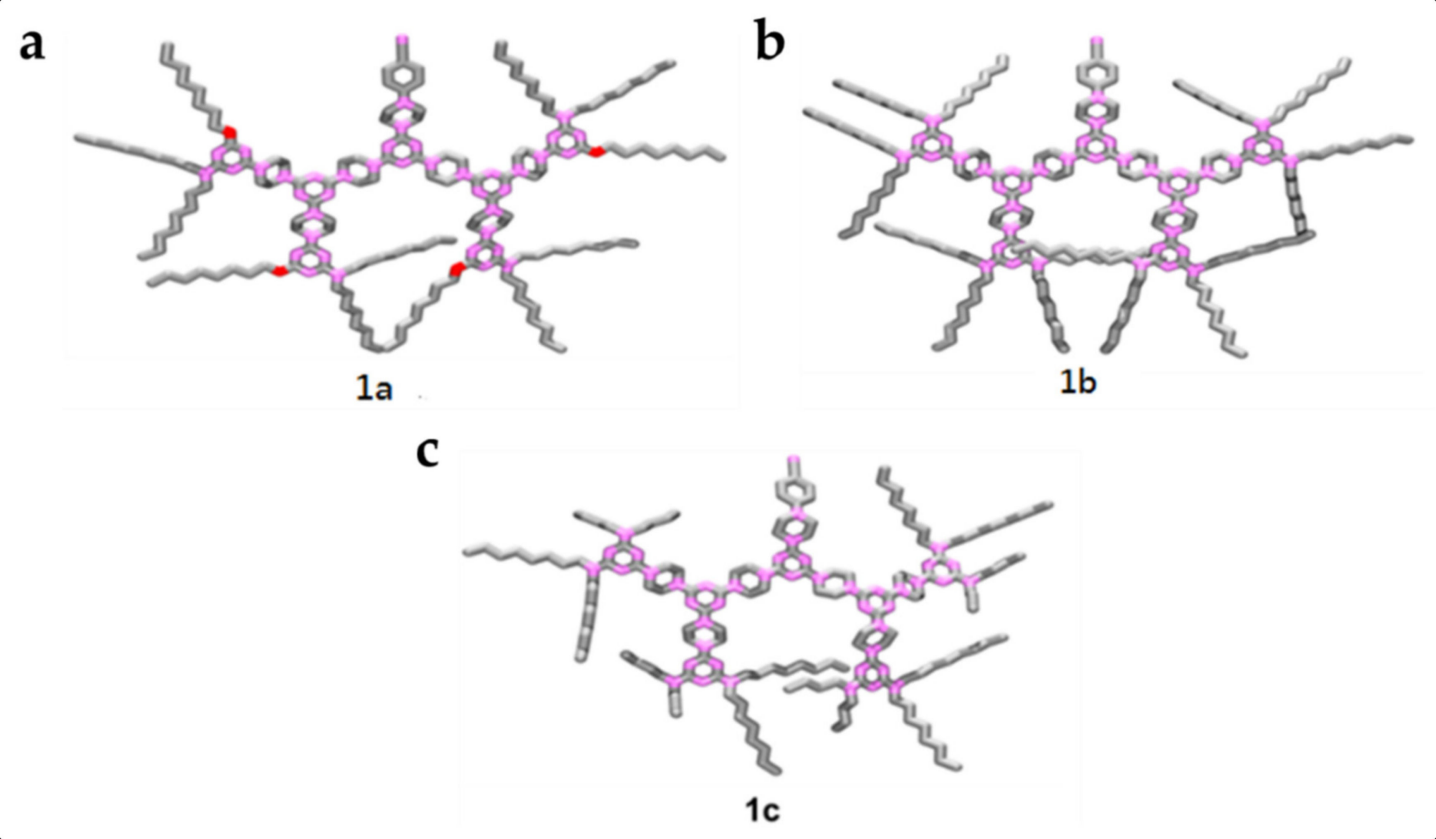
The conformations (top views) of dendrons **1a** (**a**), **1b** (**b**), and **1c** (**c**) calculated by CaChe using the MM2 model; Hs are omitted for clarity.

**Figure 5:**
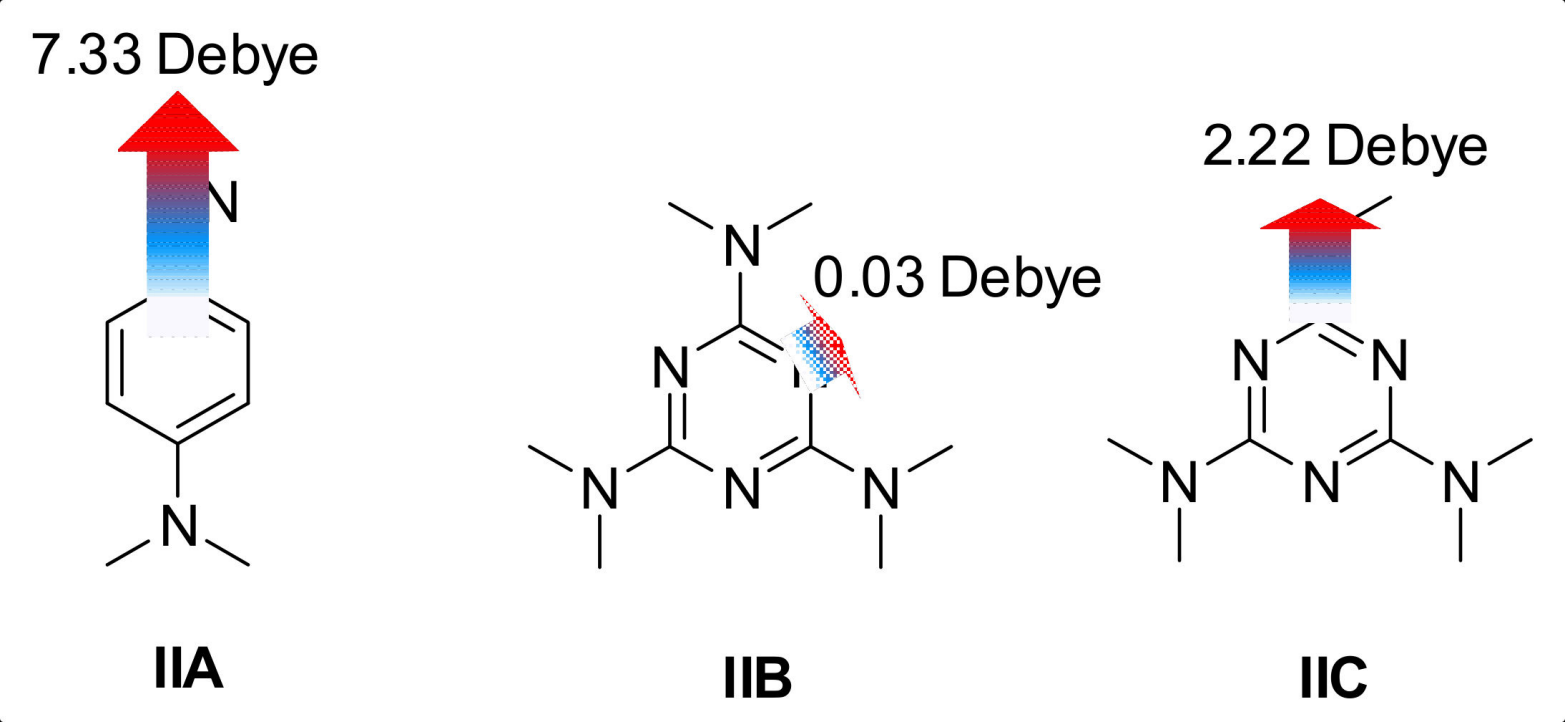
The calculated dipole moments of **IIA**-**IIC**. Arrows (red: partially negative; blue: partially positive) indicate the dipole orientations of the fragments.

The optimized geometries of dendrons **1a**–**c** were quite similar to each other in the gas phase; there were no serious steric congestion in their frameworks, and only part of alkyl group were overlapped in the peripheral moiety (Figure 4; side views: Figure S5). Such minor (almost negligible) variation between the optimized geometries of **1a**–**c** seemed to us unlikely to be responsible for the absence of any mesogenic behavior of **1a** with respect to **1b** and **1c**. We, thus, further calculated the dipole moment of each constructing unit of the dendrons. Dendrons **1a**–**c** consisted of one cyanobenzene **IIA** unit and two triazine moieties, i.e., **IIB** and **IIC** in their dendritic frameworks (Figure 5). For simplicity, the dimethylamino and methoxy moieties were used to replace dialkylamino and alkoxy groups, respectively, for molecular simulation in the gas phase. The frequency and geometry optimizations of **IIA** was first performed by the Gaussian 09 at the B3LYP/6-31G** level, and the dipole moment of **IIA** was calculated to be 7.33 Debye, which is close to that reported in the literature (7.28 Debye) [60,61,62,63]. The corresponding optimizations of **IIB** and **IIC** were, thus, accomplished in a similar manner. The dipole moment of **IIB** and **IIC** were calculated to be 0.03 and 2.22 Debye, respectively. The calculation details and the corresponding conformations of **IIA**-**IIC** are provided in supporting information (Table S1 and Figure S6).

**Figure 6:**
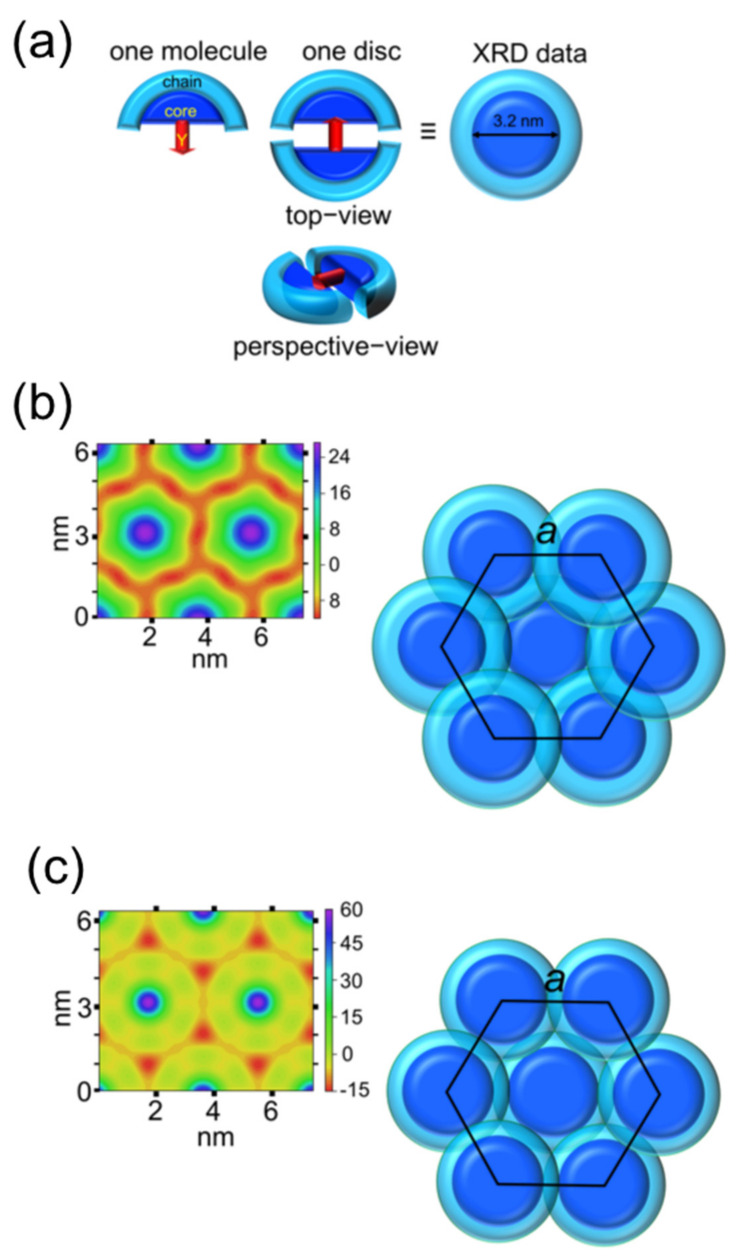
Electron density map (EDM) and scaled disc packing for **1b**,**c**. (**a**) Schematic representation of one molecule and one disc in the dimer formation model derived from XRD data; (**b**,**c**) EDMs (right) and scaled disc packing (left) of **1b** and **1c**.

**Figure 7:**
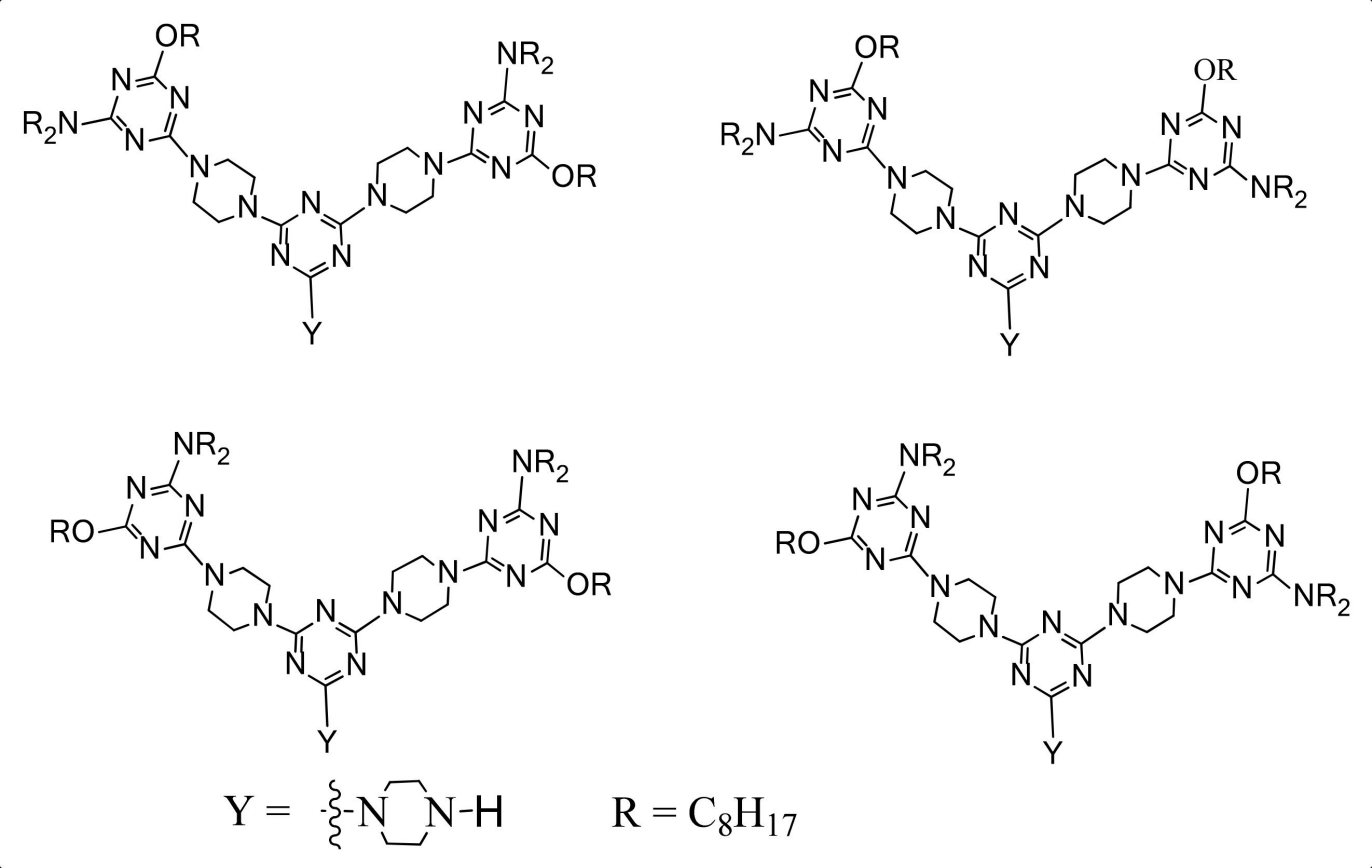
Conformational isomers of piperazine-dendrons.

With the above optimized results, we assumed that dendrons **1b** and **1c** may form a dimer in the solid stacking because of the intermolecular interaction of dipole unit **IIA**. On the other hand, electron density maps (EDM) of **1b**/**1c** in LC states were calculated from XRD data [63], and the results clearly reveal that, in these two cases, one disc is significantly larger than the dimensions of one dendron and is close to two dendrons packed with antiparallel alignment of polar -CN ends (Figure 6a). Scaled schematic representations of discs and disc packing, along with corresponding EDMs of **1b**,**c**, are shown in Figure 7. For **1b** and **1c**, columnar hexagonal packing of discs were obtained from the EDM results and structure simulations. The details for carrying out the EDM analysis and corresponding scaled schematic representations of disc/disc-packing are provided in supporting information (Figure S7). The dimeric dendron-pair in one disc of **1b** and **1c**, arising from the strong polar interaction of **IIA** between two dendronic halves, may behave as the reported dendrimers in the condensed phases and thus exhibited mesogenic phases on thermal treatment [44,45,46,47,48,49]. For dendron **1a**, it is reasonable to assume that a dimeric unit is also formed first due to the dipole moment **IIA**. However, another dipole moment from **IIC** may further increase the side-to-side interaction between dimeric dendron-pair of **1a**, thus probably leading to the non-liquid-crystalline behavior. Another possibility of non-mesogenic behaviors of **1a** on thermal treatment may arise from the lack of branching in the ether derivatives, which allows for easier crystallization. Both could be supported by the high enthalpy (52.51 kJ/mol) of **1a** between isotropic and crystalline state on cooling. Without the dipole interaction and with more branch alkyl chains at the periphery, each paired unit of dendrons **1b** and **1c** is separated because of the peripheral alkyl vibration, thus resulting in the formation of hexagonal columnar arrangement.

For **1b**, the broad XRD signals at 12.77 Å can also be attributed to the intracolumnar periodicity as the ratio of the two broad signals of 12.77 and 4.35 Å is 2.94, which is similar to that (12.13 Å/4.17 Å = 2.91) of **1c**. Therefore, these ratios suggest that, within columns, each dendron rotates 120° and repeats the same direction every four dendrons. In such packing, the monomer dipoles are also, overall, canceled.

It is worthy to note that the isotropic temperature of dendron 1b (molecular weight: ~3161) on heating was 194.0 °C, which is similar to that of **1c**, 189.9 °C (molecular weight: ~2712), and that the Iso-to-Col_h_ transition temperature of **1b** on cooling was ~191 °C, which was also close to that of **1c**, 187 °C, indicating that the stacking patterns of dendrons **1b** and **1c** are very similar in the solid state, and the variation of molecular weight for **1b** and **1c** seem not to have significant influence on the thermal behaviors around their isotropic state. However, the Col_h_-to-Cr transition temperature of **1b** was ~83 °C, which is much lower than that of 1c (~121 °C), and this may be because the peripheral groups of 1b [R^1^ = R^2^ = −N(*n*-C_8_H_17_)_2_] were more flexible than those of **1c** [R^1^ = −N(*n*-C_8_H_17_)_2_, R^2^ = −N(*n*-C_4_H_9_)_2_], resulting in the more difficulty of solidifying of **1b** on cooling. Interestingly, the molecular weight of **1a** (~2716) was close to that of **1c**, but the isotropic temperature of dendron 1a on heating was 180.8 °C, a little less than that of **1c**. Additionally, the first transition of **1a** on cooling is 159.1 °C, much lower than that of **1c**. In principle, the additional peripheral dipole moment of **1a** should result in more condense stacking in the solid state due to the side-by-side interaction, then giving a higher isotropic temperature on heating or the first transition on cooling in comparing with those of **1c**. However, the observed thermal behaviors is on the contrary to this assumption, which may be attributed to the isomeric effect of **1a** [44,46,47,49]. As shown in Scheme 1, conformational isomers of dendrons **1a** were expected to be produced from the reaction of 4-piperazinobenzontrile with **G_3a_-Cl** because **G_3a_-Cl** is generated from the combination of two equivalents of G-2 piperazine-dendron and cyanuric chloride. One piperazine-dendron consists of at least four conformational isomers, as demonstrated in Figure 7; therefore, dendrons **1a** and **G_3a_-Cl** should consist of plural isomers in their corresponding preparation, as discussed in the literature [44]. For the rationalization of liquid crystallinity of **1a**–**c**, in addition to the intermolecular dipole-dipole interactions, the bulkiness ratio of the core and alkyl portions can also be an important factor. The 16 C8-chains of **1b** as compared to 12 C8-chains result in smaller core/chain bulkiness ratio of **1a** to lead to its liquid crystallinity. For **1a** and **1c**, though the total numbers of chain carbons are the same for the two, the bulkiness of fork-like −N(C_4_H_9_)_2_ of **1c** is larger than that of the straight -OC_8_H_17_ of **1a** to lead to the core/chain bulkiness ratio of **1c** to be smaller than that of **1a** and, therefore, better liquid crystallinity of **1c**.

## 4. Conclusions

In summary, dendrons **1a**–**c** were efficiently prepared in good yields. Dendron **1a** with dialkylamino and alkoxy peripheral substituents does not exhibit any mesogenic phases on thermal treatment because of its strong side-by-side interactions from the peripheral dipole or from the lack of branching in the ether derivatives, which results in easier crystallization. However the non-mesogenic **1a** can be converted to be mesogenic **1b** or **1c** just by changing alkoxy to dialkylamino substituents. Therefore, with the vibration of peripheral alkyl group, dendrons **1b** and **1c** with only one dipole moiety (aryl-CN) in the dendritic skeleton lead their molecular arrangements to behave similar to those of reported dendritic dimers [44,45,46,47,48,49] and, thus, exhibit columnar phases on thermal treatment. In addition, the trend of bulkiness ratio of the core and alkyl portions, **1b** > **1c** > **1a**, may also play an important role to the resulting same trend of liquid crystallinity. However, the mesogenic range of dendron **1b** is broader than those of dendrimers in reported literatures [44,45,46,47,48,49], and dendron **1b**, possessing fewer synthetic steps and better yield in incorporation of CN unit in the dendritic core, therefore, can be very useful as solvating reagents in the development of opto-electronic devices in the future. 

## Data Availability

The data presented in this study are available on request from the corresponding author.

## Supplementary Materials

The following supporting information can be downloaded at: https://www.mdpi.com/article/10.3390/nano12020185/s1, Figure S1: DSC spectra of **1a**, Figure S2: DSC spectra of **1b**, Figure S3: DSC spectra of **1c**, Figure S4: POM at 136.5 °C of **1a**, Figure S5: The conformations of dendrons **1a**–**c** from the side views, Figure S6: The corresponding conformations of **IIA**-**IIC**, Figure S7: The details for carrying out the EDM analysis and corresponding scaled schematic representations of disc/disc-packing, Table S1: Computational detail.
