# Supplementary material for: Converting non-Mesogenic to Mesogenic Stacking of Amino-s-Triazine-Based Dendrons with p-CN Phenyl Unit by Eliminating Peripheral Dipole
Source: Nanomaterials (Basel). 2022 Jan 6;12(2):185. doi: 10.3390/nano12020185 (PMC8782037; doi:10.3390/nano12020185)
Supplement: Supplementary file 1 [file nanomaterials-12-00185-s001.zip › nanomaterials-1494027-supplementary.pdf]

nanomaterials

Supplementary Materials

Converting non-Mesogenic to Mesogenic Stacking of Amino-*s*-Triazine-Based Dendrons with *p*-CN Phenyl Unit by Eliminating Peripheral Dipole

Yao-Chih Lu [1], Yu-Tsz Hsu [2], Tsung-Yen Yang [1], I-Chun Liou [1], Sheng-Wei Wang [1], Po-Chia Huang [3], Jey-Jau Lee [3], Long-Li Lai [1,*] and Hsiu-Fu Hsu [2,*]

1 Department of Applied Chemistry, National Chi Nan University, No. 1 University Rd., Puli, Nantou 545, Taiwan; s103324901@mail1.ncnu.edu.tw (Y.-C.L.); s107324508@mail1.ncnu.edu.tw (T.-Y.Y.); s97324528@ncnu.edu.tw (I.-C.L.); yutze0301@gmail.com (S.-W.W.)
2 Department of Chemistry, Tamkang University, No. 151, Yingzhuan Rd., New Taipei City 251, Taiwan; s108324516@mail1.ncnu.edu.tw
3 National Synchrotron Radiation Research Center, No. 101, Hsin-Ann Rd., Hsinchu 300, Taiwan; huang.pc@nsrrc.org.tw (P.-C.H.); jjlee@nsrrc.org.tw (J.-J.L.)
* Correspondence: lilai@ncnu.edu.tw (L.-L.L.); hhsu@mail.tku.edu.tw (H.-F.H.)

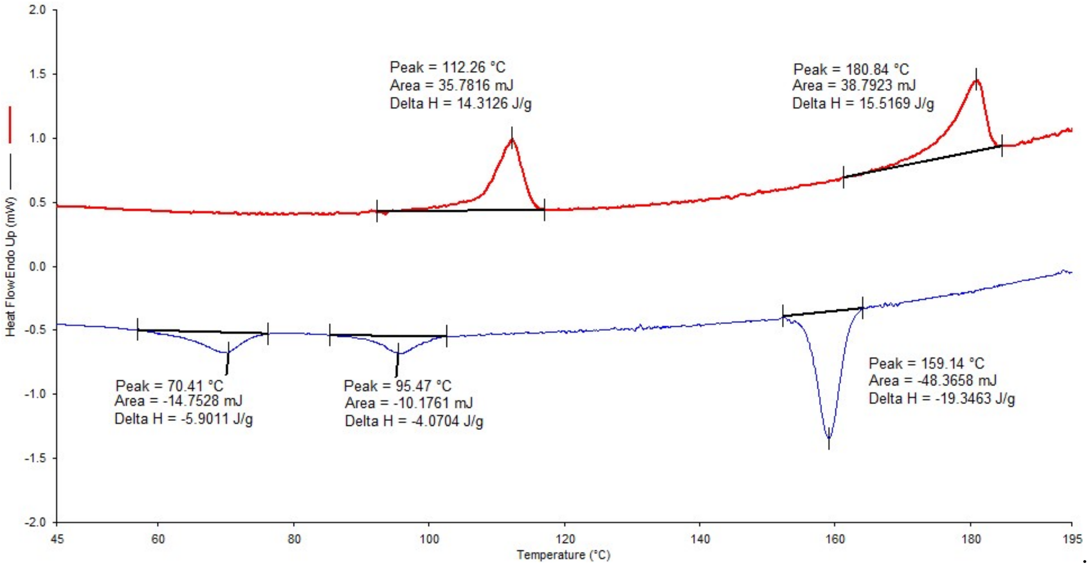

Figure S1. DSC spectra of **1a**.

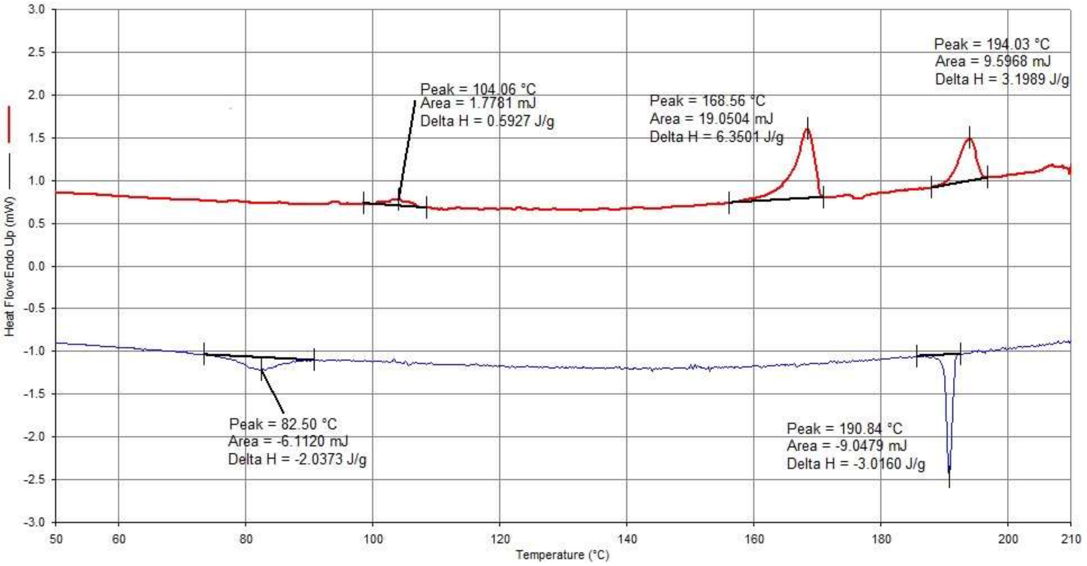

Figure S2. DSC spectra of **1b**.

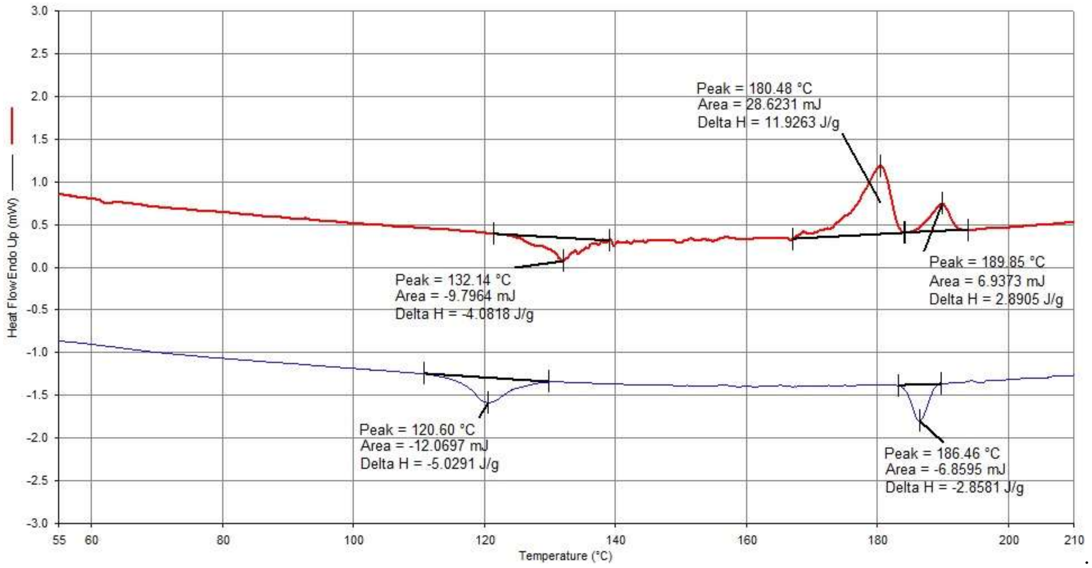

Figure S3. DSC spectra of **1c**.

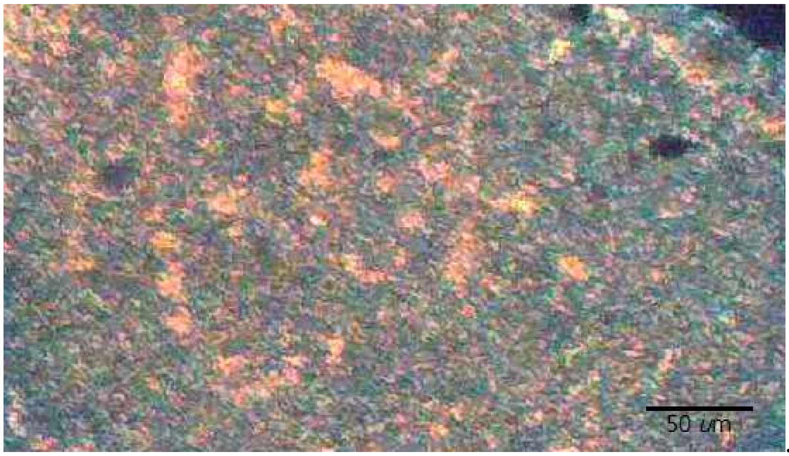

Figure S4. POM at 136.5°C and poder-XRD at 140°C of **1a**.

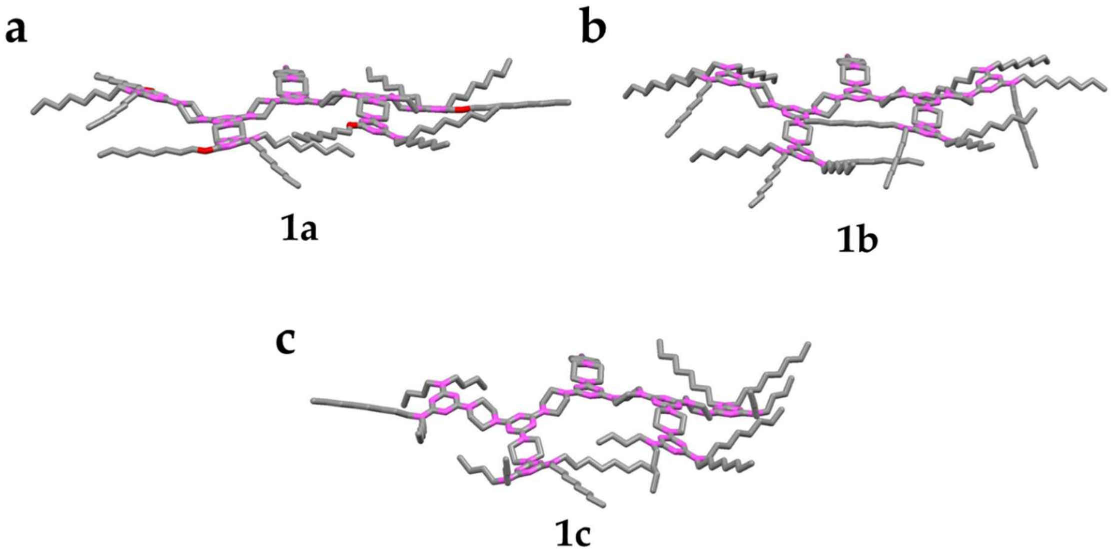

Figure S5. The conformations of dendrons **1a-1c** from the side views.

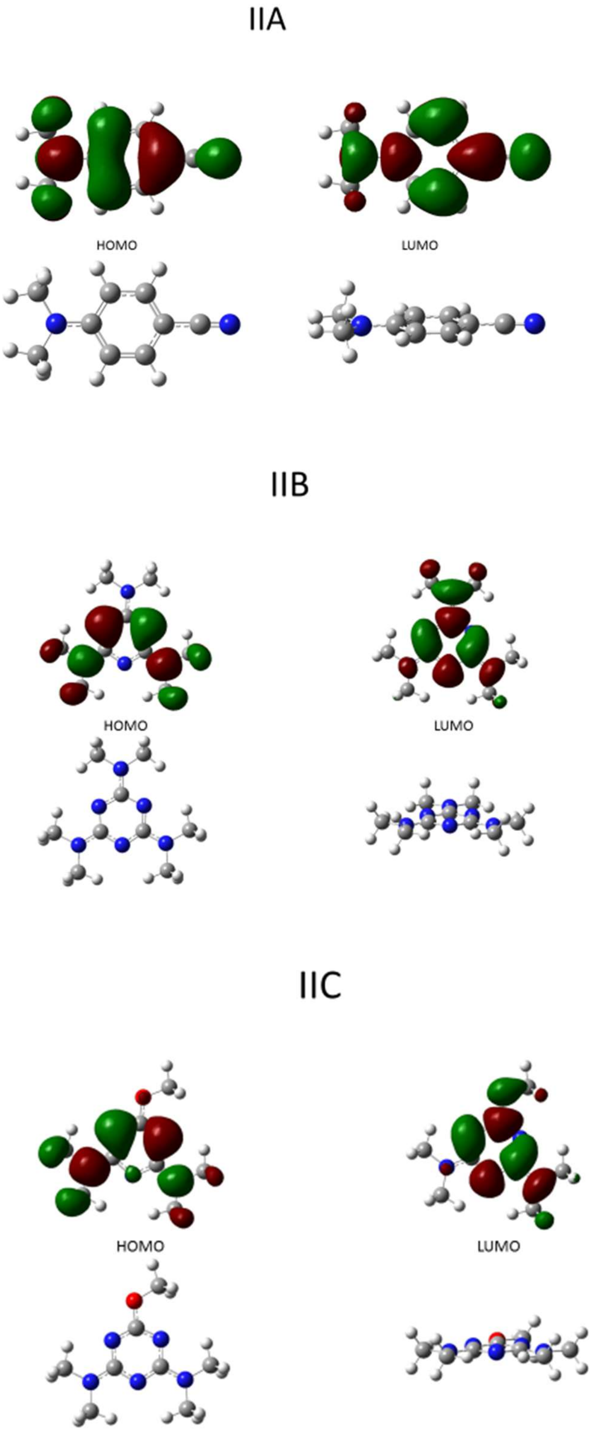

Figure S6. The conformations of **IIA–IIC** in different views and the corresponding HOMO and LUMO of **IIa–IIc**.

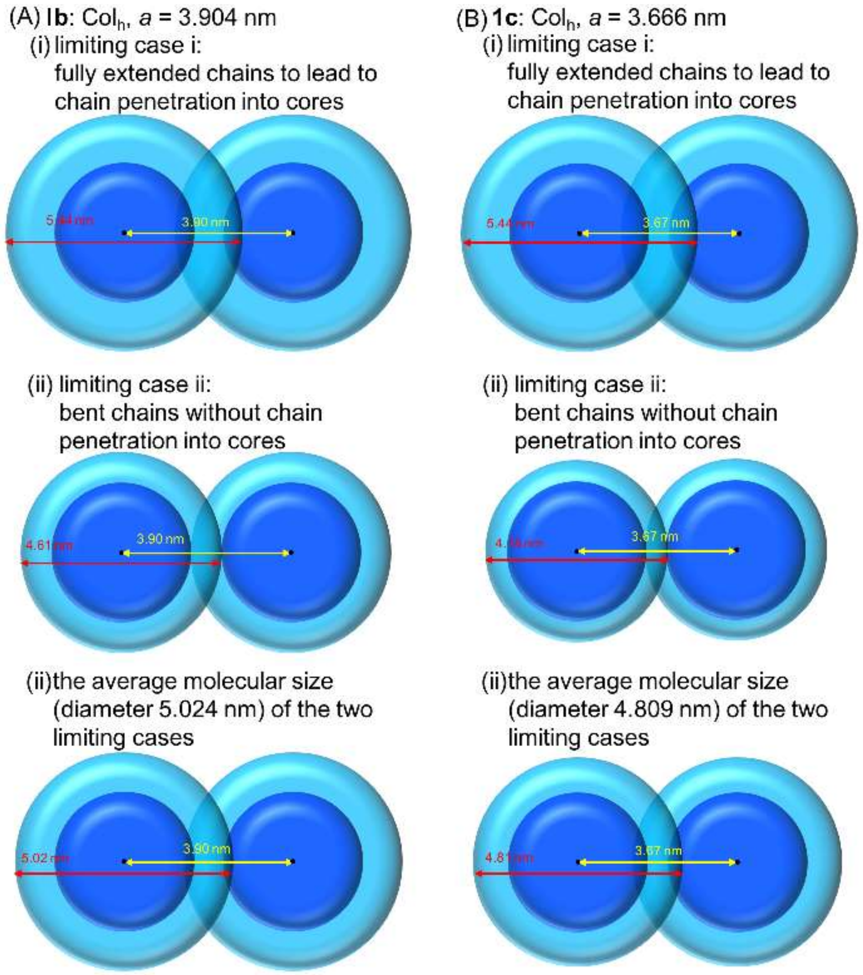

Figure S7. Estimation of molecular diameters from XRD data and optimized molecular structures for **1b** (**A**), and **1c** (**B**). For **1b** and **1c**, two limiting cases, (i) showing fully extended chains to lead to significant chain-penetration into adjacent cores and (ii) showing bent-chains without chain-penetrationinto adjacent cores. The average disc diameters of the two limiting cases for **1b** and **1c** were used in the hexagonal packing scheme in Figure EDM of the main text.

Table S1. Computational detail:

The geometric optimizations and frequencies of **IIa**-**IIc** were calculated by density functional theory (DFT) at the B3LYP/6-31G** level with Gaussian 09 Program.S1

IIa: 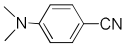

Energy : 0.16401 Hartree

Dipole moment : 7.3336 Debye

Cartesian Coordinates (Angstroms)

atom	X	Y	Z
C	-0.878579	-1.187029	-0.253034
C	-2.262519	-1.189799	-0.254384
C	-2.984683	-0.004915	-0.020412

C	-2.268880	1.178043	0.216700
C	-0.880260	1.180236	0.225574
C	-0.143600	-0.001180	-0.014766
C	-4.414533	-0.007481	-0.025132
N	-5.579051	-0.009924	-0.029642
N	1.252464	-0.008844	-0.063284
C	1.976190	1.260537	0.026392
C	1.983188	-1.120747	0.564486
H	-0.359285	-2.111877	-0.474471
H	-2.798956	-2.112192	-0.451062
H	-2.808136	2.099337	0.411322
H	-0.369933	2.109013	0.445581
C	3.393133	1.106361	-0.529403
N	4.086454	0.037853	0.184303
C	3.393832	-1.233657	-0.008615
H	2.030044	1.621208	1.067442
H	1.450458	2.009964	-0.569704
H	1.449561	-2.056066	0.398847
H	2.052236	-0.959168	1.652623
H	3.933959	2.047386	-0.383906
H	3.319983	0.921025	-1.617169
H	5.047505	-0.032247	-0.137565
H	3.933334	-2.022769	0.525803
H	3.320435	-1.529418	-1.071810

IIb: 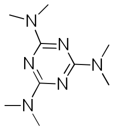

Energy : 0.20996 Hartree

Dipole moment : 0.0294 Debye

	Cartesian Coordinates (Angstroms)		
atom	X	Y	Z
N	1.224111	0.637674	-0.003044
C	0.056632	1.305892	-0.008725
N	-1.164277	0.741250	-0.007631
C	-1.159345	-0.603896	-0.004347
N	-0.059834	-1.378897	0.000659
C	1.102662	-0.702016	0.001281
N	-2.374293	-1.236461	-0.007423
N	2.258110	-1.437664	0.010841
N	0.116132	2.674364	-0.021483

C	1.376896	3.391275	0.007984
C	-1.077504	3.498149	0.009491
C	-2.491663	-2.682272	0.005486
C	-3.625787	-0.503298	0.003329
C	2.249533	-2.888213	-0.000995
C	3.568540	-0.815787	-0.000523
H	1.431816	4.095688	-0.832457
H	1.476048	3.968152	0.938327
H	2.197438	2.682153	-0.061460
H	-1.077172	4.198731	-0.835951
H	-1.956778	2.862306	-0.049490
H	-1.119773	4.087983	0.936008
H	-3.013479	-3.019943	0.911643
H	-3.068673	-3.026561	-0.863384
H	-1.499828	-3.125447	-0.022878
H	-4.233412	-0.769745	-0.872061
H	-4.207172	-0.745965	0.903531
H	-3.419314	0.563485	-0.013309
H	2.791399	-3.280748	0.870019
H	1.222877	-3.243572	0.024054
H	2.743363	-3.270515	-0.905110
H	4.120950	-1.092258	-0.909390
H	3.455762	0.264464	0.035402
H	4.156528	-1.149083	0.865180

IIc:
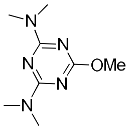

Energy : 0.21504 Hartree

Dipole moment : 2.2239 Debye

	Cartesian Coordinates (Angstroms)		
atom	X	Y	Z
N	-0.442303	-1.008080	-0.000005
C	0.859082	-0.682250	0.000008
N	1.340177	0.584582	0.000023
C	0.391309	1.516934	0.000000
N	-0.925593	1.335441	-0.000022
C	-1.293134	0.037488	-0.000013
N	-2.631233	-0.230827	-0.000015
N	1.774328	-1.695519	0.000013
C	1.377988	-3.091954	-0.000030

C	3.204426	-1.442968	0.000033
C	-3.623901	0.830512	0.000035
C	-3.150158	-1.585854	-0.000009
H	0.293352	-3.159689	-0.000119
H	1.774166	-3.602025	-0.888078
H	1.774018	-3.602051	0.888072
H	3.671612	-1.889302	0.887969
H	3.671602	-1.888973	-0.888076
H	3.382000	-0.370663	0.000223
H	-4.264623	0.752852	0.888384
H	-3.120107	1.793956	-0.000122
H	-4.264865	0.752685	-0.888122
H	-3.773276	-1.757065	0.887909
H	-3.773309	-1.757054	-0.887905
H	-2.323369	-2.291153	-0.000023
O	0.759185	2.809541	0.000002
C	2.159115	3.097884	-0.000023
H	2.648024	2.688706	-0.888452
H	2.227279	4.186624	-0.000132
H	2.648015	2.688891	0.888497

Reference

1. Gaussian 09, Revision, A.1, M.J. Frisch, G.W. Trucks, H.B. Schlegel, G.E. Scuseria, M.A. Robb, J.R. Cheeseman, G. Scalmani, V. Barone, B. Mennucci, G.A. Petersson, H. Nakatsuji, M. Caricato, X. Li, H.P. Hratchian, A.F. Izmaylov, J. Bloino, G. Zheng, J.L. Sonnenberg, M. Hada, M. Ehara, K. Toyota, R. Fukuda, J. Hasegawa, M. Ishida, T. Nakajima, Y. Honda, O. Kitao, H. Nakai, T. Vreven, J.A. Montgomery, Jr., J.E. Peralta, F. Ogliaro, M. Bearpark, J.J. Heyd, E. Brothers, K.N. Kudin, V.N. Staroverov, R. Kobayashi, J. Normand, K. Raghavachari, A. Rendell, J.C. Burant, S.S. Iyengar, J. Tomasi, M. Cossi, N. Rega, J.M. Millam, M. Klene, J.E. Knox, J.B. Cross, V. Bakken, C. Adamo, J. Jaramillo, R. Gomperts, R.E. Stratmann, O. Yazyev, A.J. Austin, R. Cammi, C. Pomelli, J.W. Ochterski, R.L. Martin, K. Morokuma, V.G. Zakrzewski, G.A. Voth, P. Salvador, J.J. Dannenberg, S. Dapprich, A.D. Daniels, Ö. Farkas, J.B. Foresman, J.V. Ortiz, J. Cioslowski, D.J. Fox, Gaussian, Inc., Wallingford CT, **2009**.
